# Antibiotic use, resistance development and environmental factors: a qualitative study among healthcare professionals in Orissa, India

**DOI:** 10.1186/1471-2458-10-629

**Published:** 2010-10-21

**Authors:** Krushna Chandra Sahoo, A J Tamhankar, Eva Johansson, Cecilia Stålsby Lundborg

**Affiliations:** 1 Division of Global Health (IHCAR), Department of Public Health Sciences, Karolinska Institutet, Nobels väg 9, SE 171 77 Stockholm, Sweden; 2 Department of Environmental Medicine, R.D. Gardi Medical College, Ujjain 456 006, India; 3 Indian Initiative for Management of Antibiotic Resistance (IIMAR), N.G. Acharya & D.K. Marathe College, Chembur, Mumbai, 400 071, India; 4 The Nordic School of Public Health, Gothenburg, Sweden

## Abstract

**Background:**

Antibiotic resistance is a major public health problem affecting both current and future generations. The influence of environmental factors on antibiotic use and resistance development in bacteria is largely unknown. This study explored the perceptions of healthcare providers on antibiotic use and resistance development in relation to environmental factors i.e. physical, natural, social and behavioural factors.

**Methods:**

A qualitative interview study was conducted using face-to-face, semi-structured interviews among registered allopathic doctors, veterinarians and drug dispensers in Orissa, India. The interview transcripts were analyzed using latent content analysis.

**Results:**

The main findings of this study relate to two themes: 'Interrelationship between antibiotic use, resistance development and environment' and 'Antibiotic management contributing to the development and spread of resistance'. The interviewees viewed the following as possible contributors to antibiotic use/misuse and resistance development: changes in the natural and physical environment i.e. climate variability, pollution, physiography and population growth; the socioeconomic environment affecting health-seeking behaviour and noncompliance with medication; a lack of healthcare facilities and poor professional attitudes; and ineffective law enforcement regarding medicine dispensing and disposal.

**Conclusions:**

Generally, the interviewees perceived that although behavioural and social environmental factors are major contributors to resistance development, changes in the physical and natural environment also influence development of antibiotic resistance. The respondents also perceived that there is a lack of information about, and poor awareness of, what constitutes prudent use of antibiotics. They suggested a need for information, education, dissemination and proper implementation and enforcement of legislation at all levels of the drug delivery and disposal system in order to improve antibiotic use and prevent pharmaceutical contamination of the environment.

## Background

The environment is an omnipresent factor governing all human and animal activities and behaviours and also the micro-organisms that cause diseases in them. According to Smith et al. the environment consists of four major components-physical, natural, social and behavioural [1]. The physical environment is the external surroundings and conditions such as temperature, humidity, rainfall etc. The natural environment encompasses the geographical areas along with all living and non-living components. The social and behavioural components include cultural, socio-political and contextual factors as well as individual behaviour. Globally, an estimated 24% of the disease burden and 23% of all deaths can be attributed to environmental factors [2]. However, the importance of these factors as modifying components of human and animal medicine is not always fully recognized. Further, in general, there is a paucity of information on the influence of environmental factors on antibiotic use and development of antibiotic resistance.

All over the world, development of resistance to antibiotics is on the increase amongst pathogenic bacteria [3,4]. However, to counter the situation, very few new antibiotics have come into use in the last three decades [5] and thus there is a need to conserve the antibiotics we already have.

In general in India, and also in Orissa, there is a high incidence of infectious diseases, increasing use of antibiotics by a wide range of health care providers and increasing resistance, associated with commonly occurring indiscriminate use of antibiotics in humans as well as in animals [4,6,7]. In addition to this, in case of Orissa, which is an economically underdeveloped state compared to many other states of India, there exists a large segment of population with economic incapability to use appropriate antibiotics, a lack of an adequate health care system and a pronounced vulnerability to weather related changes due to its geographical location [8].

It is possible that environmental factors have a role to play in the way antibiotics are used in a population and the consequent development of resistance. To explore this issue, a qualitative study is needed to understand the way people perceive the issue and the meaning they attach to it, which can be further addressed quantitatively.

We therefore, conducted a qualitative study among healthcare providers in the eastern Indian state of Orissa, taking it as an example, to explore their opinions about antibiotic use and antibiotic resistance development in relation to environmental factors.

## Methods

### Study setting

Out of the 30 districts of Orissa, four were purposely selected for the study. These districts were selected based on the geography, population density, urbanization, ethnicity, literacy rate and availability of healthcare facilities. The intention was to incorporate a variety of behavioural, social, natural and physical environmental factors. The districts of Cuttack and Khurda, were selected as representing an urban environment, proximity to sea, high population density (about 640 people per square kilometre), and high literacy rate (about 80%) [9]. The districts of Koraput and Malkangiri, were selected to represent inland rural regions with forests, hills and tribal areas, a low population density (about 120 people per square kilometre), and a low literacy rate (about 40%) [9]. The health system in both the set ups incorporated diverse components - health care providers belonging to public, private and nonprofit organizations sector incorporating practitioners of allopathic and the AYUSH system i.e. practitioners of Ayurveda, Yoga and naturopathy, Unani, Siddha and Homoeopathy [10]. There were also untrained allopathic prescribers, generally referred to as 'quacks' and local traditional faith healers. Amongst the two set ups private healthcare is more common in urban areas than in rural areas and also the urban areas have more predominance of registered allopathic and AYUSH doctors and less of quacks and traditional faith healers.

### Participants

Only allopathic providers were selected as they were most likely to handle antibiotics. To maintain a balanced distribution among the various types of healthcare providers i.e. registered allopathic doctors, veterinarians and drug dispensers included in the study, two participants from each district and from each healthcare provider category (total 24) were selected. Thirty-six potential participants had to be contacted to reach the desired distribution among the participants, as 12 (seven registered allopathic doctors, three veterinarians and two drug dispensers) did not agree to participate due to their busy schedule. All the participants were contacted in person or by telephone by the first author (KCS) before the interviews. All the participants participated voluntarily; no payment was offered nor given. Detailed information about the interviewees is presented in Table 1.

**Table 1:** Study area and background characteristics of interviewees

Interviewees	Study area - Orissa, India							
	Rural districts				Urban districts			
	
	Malkangiri		Koraput		Cuttack		Khurda	
**Registered allopathic doctors (M)** Sex (female (f) or male (m)) Age (experience) in years Qualification	1 f 31 (3) MD	2 m 29 (3) MBBS	3 f 27 (2) MBBS	4 m 47 (21) MS	5 m 65 (41) MD	6 m 72 (40) MS	7 m 53 (28) MS	8 m 51 (28) MD
**Veterinarians (V)** Sex (female (f) or male (m)) Age (experience) in years Qualification	1 m 54 (30) MVSc	2 m 30 (5) BVSc	3 m 71 (37) BVSc	4 m 50 (27) MVSc	5 m 50 (30) MVSc	6 m 38 (10) MVSc	7 m 55 (32) MVSc	8 m 48 (25) BVSc
**Drug dispensers (D)** Sex (female (f) or male (m)) Age (experience) in years Qualification	1 m 34 (8) B.Pharm.	2 m 29 (4) Dip.Pharm	3 m 50 (34) B.Pharm.	4 m 39 (12) Dip.Pharm	5 m 44 (17) B.Pharm.	6 m 27 (5) B.Pharm.	7 m 34 (10) Dip.Pharm	8 m 35 (7) B.Pharm.

M-Registered allopathic doctors, V-Veterinarians and D-Drug dispensers, MBBS-Bachelor in Medicine and Bachelor in Surgery,

MD-Master in Medicine, MS-Master in Surgery, MVSc-Master of Veterinary Science, BVSc-Bachelor of Veterinary Science,

B. Pharm.-Bachelor of Pharmacy, Dip. Pharm.- Diploma of Pharmacy

### Data collection procedures

Data was collected by face-to-face interviews using open-ended questions (Appendix 1). All interviews were conducted in a mix of local language (Oriya) and English and lasted for an average of 29 (range 20-40) minutes for registered allopathic doctors, 24 (range 15-40) minutes for veterinarians and 17 (range 13-20) minutes for drug dispensers. All the interviews were conducted by the first author who is a native of the study area and has an educational background in biological and environmental sciences as well as experience in social services. The co-investigators have different backgrounds: agriculture, environmental medicine, pharmacy, nursing and qualitative research, all with a public health perspective.

### Data management and analysis

The tape-recorded interviews were transcribed verbatim and the transcripts were translated into English and analyzed using latent content analysis [11].

Latent content analysis deals with the identification of the underlying meaning of the text. The selection of the unit of analysis is the first step in the content analysis. Meaning units i.e. parts of the original transcript text that are related to the aim of the study are selected from the interview transcript. The meaning units are condensed and coded. Similar codes are clustered together and collapsed into subcategories and categories. A category is a group of related codes. The theme is then identified and illustrates the underlying meaning of the text.

Such a procedure was followed to analyse the contents of the interviews. During the conduct of this procedure, the tape-recorded versions and the Oriya and English transcripts were consulted repeatedly during the coding procedure to understand the full meaning of the text.

### Ethical approval

The study was approved by the ethical committee of the Kalinga Institute of Medical Sciences, Orissa, India. Before the interview, information was given about the purpose of the study to the interviewees and they were told that they could withdraw from the study at any time. Written consent was obtained from each interviewee. All the interviews were carried out in complete privacy at the place chosen by the interviewee (residence, hospital, clinic, drug store) and the names of the interviewees were kept confidential.

## Results

Two major themes 'Interrelationship between antibiotic use, resistance development and environment' (Table 2) and 'Antibiotic management contributing to the development and spread of resistance' (Table 3) emerged from the analysis. The findings are presented under each major theme/category/subcategory with quotes from the respondents at the end of the each paragraph. Information by the authors in the quotations is presented in square brackets.

**Table 2:** Theme 'Interrelationship between antibiotic use, resistance development and environment'

	Interrelationship between antibiotic use, resistance development and environment			
**Categories**	Influence of ecological factors on antibiotic prescription		Influence of presence of antibiotics in the natural environment leading to resistance development	
**Sub categories**	Abiotic factors	Biotic factors	Antibiotics in the environment	Effect of presence of antibiotics in the environment
**Codes**	Physiography Seasons High temperature Pollution	Biotopes Population density	Water Terrain Air	Micro organisms Aquatic organisms Grazing animals Rural people Resistance

**Table 3:** Theme 'Antibiotic management contributing to the development and spread of resistance'

	Antibiotic management contributing to the development and spread of resistance				
**Categories**	Antibiotics use and factors influencing prescribing		Health systems and policy factors influencing resistance		
**Sub-categories**	Humans	Animals	Patients	Health systems and professionals	Policy and regulatory issues
**Codes**	Broad and narrow spectrum "Higher" antibiotics High loads of patients Prior prescribers Unqualified medical providers Influence of pharmacy company	Therapeutic uses Prophylactic uses Antibiotics as growth promoter Uses in poultry Uses in dairy farming	Noncompliance Self-medication Poverty Ignorance Lack of awareness	Inadequate prescription Irrational use Improper diagnosis Lack of infrastructure	Fake and Low-quality medicine Without prescription Weak implementations of policy

### Interrelationship between antibiotic use, resistance development and environment

This theme evolved from two categories (i) Influence of ecological factors on antibiotic prescription and (ii) Influence of presence of antibiotics in the natural environment leading to resistance development.

#### Influence of ecological factors on antibiotic prescription

Under this category two main subcategories were identified (i) Abiotic factors (ii) Biotic factors

##### Abiotic factors

The abiotic factors are the non-living factors of the environment e.g. chemical-minerals, water; physical-temperature, sunlight. Two main abiotic factors, climate and physiography, were identified in this subcategory. Interviewees perceived that climatic factors such as precipitation, temperature and humidity had significant impact on antibiotic prescription. Interviewees felt that seasons influenced the occurrence of infectious diseases and hence changed the antibiotic prescription patterns. According to them, each season had its own types of diseases. In high temperature conditions in the summer, stress-related diseases in animals were more common. Diarrhoea was more common in humans in the rainy season. Both complaints led to high antibiotic prescription. High temperatures in the summer and high humidity in the rainy season resulted in more skin infections in humans and in the winter, although people were normally healthier, respiratory tract infections, including the common cold, led to misuse of antibiotics.

"When the monsoons start, at that time bacterial disease is more common and it is common in animals. There is less infection in winter. In summer, protozoan diseases are more common, due to summer stress; all Indian animals carry most of the diseases. Protozoan diseases are particularly common in forest areas. In coastal areas, a higher antibiotics prescription rate is more common. The environment is slowly changing, the temperature is rising." (Veterinarian no. 8, BVSc, 25 years experience)

"In most of the cases, high doses of antibiotics are prescribed from the beginning, probably due to the change in climate." (Drug dispenser no. 1, B.Pharm., 25 years experience)

The interviewees' perception was that antibiotic use varied in different geographical areas, for example - in coastal and non-coastal areas. According to prescribers, higher antibiotics were used in coastal areas to control infectious diseases whereas in hilly areas most of the patients took traditional herbal treatment and lower antibiotics. They viewed that geographical variation in antibiotic consumption and resistance may be associated with factors such as regional climate, socioeconomic conditions, local population density and behaviours of a particular community towards antibiotic prescribing or consuming.

"In my view rampant use of antibiotics for minor infections is one cause of resistance in coastal areas; they are prescribing higher antibiotics unnecessarily." (Registered allopathic doctor no. 3, MBBS, 2 years experience)

The interviewees also felt that pollution had a significant impact on antibiotic prescription. They perceived that in polluted areas, both infections and resistant bacteria spread more quickly.

"In polluted areas resistance is high, where pollution [air, water] is more, infections spread quickly. Now higher antibiotics are used due to pollution. In polluted areas, antibiotics are used for many days. We have to change two or three antibiotics." (Registered allopathic doctor no. 1, MD, 3 years experience)

##### Biotic factors

According to the interviewees, the health of humans is influenced by their habitat i.e. the area where they live and its components like plants, animals, micro-organisms and other human beings. The interviewees further perceived that large numbers of people making use of fewer health care facilities, leading to a high patient load in hospitals, caused improper diagnosis and inappropriate use of antibiotic treatment. The participants reported that overcrowding created unhygienic conditions which also caused infectious diseases to spread more rapidly, leading to increased irrational antibiotic prescription.

"In crowded areas, due to the unhygienic environment; infectious diseases are more common, if population density is high, then there are more diseases and more antibiotic prescriptions." (Registered allopathic doctor no. 5, MD, 41 years experience)

#### Influence of presence of antibiotics in the natural environment leading to resistance development

Two main subcategories were identified under this category (i) Antibiotics in the natural environment and (ii) Effect of presence of antibiotics in the natural environment.

##### Antibiotics in the natural environment

Interviewees put forward a viewpoint that, due to the widespread improper disposal of pharmaceuticals, antibiotics are contaminating the aquatic as well as the terrestrial environment. According to them, hospital waste, out-of-date medicines from shops and unwanted household pharmaceuticals were not disposed properly and this was polluting water, grasslands and air. They believed that properly implemented and enforced environmental law or policy was essential for the safe disposal of pharmaceutical waste.

"Out-of-date medicine, if thrown outside, will impact the environment. What we throw away, it will mix with water, again when we drink that water, that water again enter into the blood and obviously it will have an impact. Environmental law is essential." (Registered allopathic doctor no. 7, MS, 28 years experience)

##### Effect of presence of antibiotics in the natural environment

According to the interviewees, the improper disposal of antibiotics was affecting aquatic organisms, grazing animals and also human beings, who were using river or pond water for drinking and cooking.

"If pharmaceutical wastes are disposed improperly, it will affect. Expired medicine loose its efficacy, but not toxicity.... If we throw these wastes outsides that will mix up with pond or river water; if that water is taken by human or animal it may create problem; what happened recently at Cuttack, some cattle died by eating expired medicine including some antibiotics which were thrown improperly at the river beach. Some aquatic organisms also died. Every drug is in proper dose medicine and improper dose poison." (Registered allopathic doctor no. 5, MD, 41 years experience)

Interviewees also assumed that antibiotics in the environment had a significant impact on micro-organisms. One interviewee pointed out that waterborne bacteria might develop resistance if they were exposed to low doses of antibiotics in water.

"If waterborne diseases causing bacteria are exposed to low doses of antibiotic every day, they gradually become tolerant to that particular drug and resistance will develop." (Veterinarian no. 6, MVSc, 10 years experience)

Some of the interviewees were of the opinion that there was not much research on the environmental aspects of antibiotic resistance and they suggested further research in this area in order to find out the exact problems and their proper solutions.

"Antibiotic resistance in relation to the environment is a research question, further study is essential in this field." (Veterinarian no. 4, MVSc, 27 years experience)

### Antibiotic management contributing to the development and spread of resistance

This theme emerged from the categories (i) Antibiotic use and factors influencing prescribing and (ii) Health systems and policy factors influencing resistance

#### Antibiotic use and factors influencing prescribing

Under this category, two subcategories were identified (i) Humans and (ii) Animals

##### Humans

The interviewees used the term 'higher antibiotics' in varying meanings including new generation, broad spectrum, high dose or high-price antibiotics. Their view was that the prescribing of any type of 'higher antibiotics' in the first encounter was one of the reasons for bacterial non-response to 'old' antibiotics. Most of them viewed that the indiscriminate use of antibiotics has lead to the resistance problem. In their opinion, high patient load, prior prescription by unqualified prescribers and high prices of antibiotics are major reasons for inappropriate prescribing of antibiotics. The interviewed doctors said that a single doctor prescribes to approximately 50-90 patients per day in both rural and urban hospitals. This high patient load can cause improper diagnosis, leading to inappropriate treatment and improper use of antibiotics. All the interviewed doctors mentioned that before visiting an authorized medical practitioner, many patients take earlier treatment from 'quacks', i.e. untrained people, who act as physicians and give medical advice and treatment. Furthermore, patients may also have bought medicines directly from the drug store on the advice of drug dispensers or based on their own diminutive knowledge. The interviewees said that quacks are prescribing incomplete courses and doses of antibiotics and some of them suggested that the quacks should receive training.

"Doctors are not available, quacks are helping the patients...... in my view we have to give training to them and license them, something that has recently been done in Kolkota [a city in India], and we have to train them as paramedical practitioners." (Registered allopathic doctor no. 6, MS, 40 years experience)

Drug dispensers said that sometimes prescribers are influenced by a particular company and prefer to prescribe that company's medical products. They thought that these factors influence antibiotic prescriptions. On probing, some of the drug dispensers admitted recommending antibiotics themselves even for the common cold.

"If the patients say that they have a sore throat, we prescribe five tablets of roxithromycin. We force them to take [buy] the full course, but they often just take for one day or one to two tablets. The common cold is very common here and people usually take anticold tablets with antibiotics. We prescribe lower antibiotics like cefadroxil for colds. If they aren't cured, we prescribe higher antibiotics like cefixime." (Drug dispenser no. 3, B.Pharm., 34 years experience)

##### Animals

According to the interviewed veterinary surgeons, penicillin, amoxicillin, cloxacillin, ciprofloxacin and tetracycline are the most commonly used antibiotics on dairy and poultry farms and for household pets for therapeutic purposes. These are also commonly prescribed antibiotics in humans. In their opinion, prior prescription by unqualified prescribers was influencing antibiotic prescriptions for animals although pharmaceutical companies do not exert the same influence in veterinary practice as in human medicine. Prescribers perceived that if antibiotics were used on dairy farms during lactation, it would get into the milk. In their perception, this might be one of the reasons behind the resistance development as people use a lot of milk in food preparation. All the veterinarians said that in the case of poultry, antibiotics were used both for prophylactic purposes and as growth promoters in feeds. They disliked the use of antibiotics as growth promoters and some of them suggested the use of probiotics for this purpose instead.

"In poultry, antibiotics are generally used as growth promoters in feeds, e.g. tetracycline and lincomycin. Nonhuman use of antibiotics definitely creates resistance problems in humans." (Veterinarian no. 6, MVSc, 10 years experience)

#### Health systems and policy factors influencing resistance

Three major factors influencing resistance were recognized under this category: (i) Patients (ii) Health systems and professionals and (iii) Policy and regulatory issues.

##### Patients

Interviewees felt that patients' noncompliance with prescribers' instructions, self-medication, poverty, ignorance about antibiotic use and lack of awareness about resistance were all possible contributors to resistance development. According to them, poverty in rural areas and self-medication in urban areas were the major reasons for patients taking incomplete courses of antibiotics. The interviewees felt that the cost of the drugs varied from company to company and believed that many patients were under the impression that more expensive drugs were more effective and were therefore purchasing high-priced antibiotics but not completing the entire course of treatment due to the high expense involved. Interviewees were of the opinion that a lack of public awareness about the consequences of improper use of antibiotics was a cause of irrational antibiotic use, which led to resistance development.

"If patients have taken incorrect medicine, it will develop resistance and due to resistance to previously prescribed antibiotics, the doctor will prescribe new, more expensive antibiotics. Because of the high price involved, the patient is unable to buy the full course, which will once again create resistance to the new antibiotics, so public awareness is essential." (Drug dispenser no. 4, Dip.Pharm., 12 years experience)

##### Health systems and professionals

A majority of the prescribers interviewed considered that a lack of infrastructure, improper diagnosis of diseases, inadequate prescription by some prescribers and irrational use of antibiotics by some patients were the major health system and professional factors responsible for improper use of antibiotics. For them, inadequate prescription meant insufficient dose or too short a course of antibiotics and irrational use involved indiscriminate and improper treatment of diseases. They said that sometimes antibiotics were used for a disease condition that might not need antibiotic treatment and this was the result of improper diagnosis.

"The common cold is a viral disease but we are prescribing antibiotics for this, possibly to prevent a suspect secondary infection, not for the original viral infection." (Registered allopathic doctor no. 3, MBBS, 2 years experience)

According to the interviewees, there was a lack of good laboratories for culturing and sensitivity testing, which caused improper diagnosis and hence irrational antibiotic prescribing in humans. Veterinarians reported that the lack of good laboratories and staff was the major factor responsible for improper use of antibiotics in veterinary practice. All interviewees considered that this erroneous way of antibiotic treatment was one of the causes of resistance development.

##### Policy and regulatory issues

Many interviewees said that weak implementation of prevailing legislation governing the health care system was one of the possible contributors to resistance development. Due to weak drug policy, fake and low-quality antibiotics are available in the market and prescription-only drugs are also available without a doctor's prescription. Some interviewed doctors suggested that the selling of antibiotics without a prescription from an authorized medical prescriber should be banned and that a strong drug policy was needed to promote rational use of drugs. The participants informed that antibiotics are also commonly prescribed by some of the homeopathic and ayurivedic healthcare providers although they are not supposed to do so. Most of the doctors suggested that antibiotics used in humans should not be used in animals.

"Now fake medicines are available in India, I do not know how it is possible." (Registered allopathic doctor no. 8, MD, 28 years experience)

"Unauthorized medical practice should be banned, either they are quacks or homeopathic or ayurivedic doctors, they should prescribe their own medicine, the majority of them are prescribing antibiotics, they don't have enough knowledge about dosage, course......half of the drugs which are banned in developed country end up and are prescribed here." (Registered allopathic doctor no. 4, MS, 21 years experience)

## Discussion

To the best of our knowledge, this is the first qualitative study exploring perceptions about the interrelationships between antibiotic use, resistance development and environmental factors, i.e. physical, natural, social and behavioural factors. The views put forward here suggest that the possible contributors to antibiotics use/misuse and resistance development are - changes in the natural and physical environment, i.e. climate variability, pollution, physiography and population growth; socioeconomic environmental factors affecting health-seeking behaviour and noncompliance with medication; a lack of healthcare facilities and poor professional attitudes; and ineffective law enforcement in relation to drug dispensing, manufacture and disposal.

### Changes in the natural and physical environment

According to Brown [12], there is a need to conduct qualitative studies in the area of environmental health to further understand how people perceive the relationship between environment and public health. Participants in our study perceived that ecological imbalances triggered by seasonal changes in weather conditions, such as temperature, precipitation and humidity; and pollution were responsible for the emergence and the re-emergence of diseases, which in turn influenced antibiotic prescriptions and antibiotic use. Our respondents perceived a seasonal pattern of occurrence of diseases e.g. skin infections and hence expected antibiotic prescriptions also to follow the same pattern. That infectious diseases show a seasonal pattern is well known. Models have demonstrated that small seasonal changes in host or pathogen factors may be sufficient to create large seasonal effects on disease incidence, which might be important particularly in the context of global environmental changes [13].

There is a general lack of studies on whether changes in weather conditions modify levels of antibiotic resistance. However, some studies from Europe and the United States have reported on geographical variation in antibiotic consumption and resistance, taking into account the influence of social and climatological factors [14-17], which was also mentioned by the participants in our study. If more studies are taken up in this area of research, and if they establish a clear-cut relationship between weather/seasonal changes, and occurrence of diseases, antibiotic prescriptions, and antibiotic resistance, although we cannot control weather or seasons, in anticipation of the impending calamities, governments and health systems can keep themselves ready with contingency plans to take up appropriate remedial/interventional measures.

Previous studies have shown that outdoor environmental air pollution from various sources, such as motor vehicles, industry, and neighbourhood-level solid waste burning, is associated with increased morbidity and mortality from respiratory infections in children and adults [18,19]. Increased morbidity might lead to increased antibiotic prescribing, which in turn might lead to increased resistance. This kind of thoughts may have been in the minds of our participants, when they brought into discussion pollution as an environmental factor resulting into more infections, more prescriptions, and more resistance.

The impact of antibiotic use on the environment was also perceived by healthcare providers. One participant suggested that antibiotic contamination of the aquatic environment may be one of the reasons for the resistance found in waterborne bacteria, due to exposure to below-threshold concentrations in water. Studies have shown that antibiotics and antibiotic resistant bacteria are found in the aquatic environment [20,21], however, very few studies [22] have looked at the impact of antibiotics in the aquatic environment on human health. Our interviewees believe that more studies in this area are essential from a public health perspective.

### Socioeconomic environment and health-seeking behaviour

Participants in this study perceived that socioeconomic and behavioural factors, i.e. poverty; self-medication and non-compliance result in inappropriate use of antibiotics. This finding is consistent with the results of other studies from low- and middle-income countries [23,24]. The practice of discontinuing antibiotics when a person feels better has been reported from various countries e.g. United Kingdom (UK) [25]. A study in Nagpur, India [26] found that constraints of poverty is the major driving force for self-medication with antibiotics. It was not considered necessary to get a new prescription and thus pay a consultation fee when a doctor had previously recommended the drug for a similar complaint [26]. The conclusion of the study was that it is important to improve physician and consumer knowledge and behaviour to use antibiotic drugs more rationally. Our study, which took place in Orissa, a socioeconomically more disadvantaged area in comparison to other Indian states, also suggests that poverty is the major driving-force for use of leftover medicines, incomplete courses and self-medication with antibiotics. On the other hand, a comparative study from eleven (economically advanced) European countries [27] indicates that a lack of awareness and an attitude towards situational use of antibiotics are contributors for irrational use of antibiotics.

### Healthcare facilities and professional attitudes

According to previous studies from India by Basu et. al. [28] and Patel et. al.[29], poor socioeconomic status, overcrowding of patients, inadequate prescription, overprescribing and improper selection of antibiotics are the major reasons behind resistance development, which was also viewed by our interviewees. Apart from this, our participants also perceived that improper diagnosis and inadequate prescription due to a lack of infrastructure and irrational prescription by unauthorized practitioners are also responsible for resistance development. This observation is similar to the findings of Kumar et. al. [30], who found that healthcare facilities with better infrastructure and prescribers with higher education and specialisation are associated with low antibiotic prescription. A previous study in UK showed that education on antibiotic prescribing and resistance at undergraduate and graduate education level is likely to enhance the quality of antibiotic prescribing [31]. The participants in our study suggested that as unauthorized practitioners serve patients in remote areas, where the availability of trained prescribers is virtually nonexistent, training them might benefit public health.

The participants also offered a view that monitoring resistance patterns at the local level and making available this data to local prescribers may help appropriate empirical therapy. They also felt that information about and awareness of what constitutes prudent use of antibiotics given to practitioners routinely, might help reduce unnecessary antibiotic use and hence prevent resistance development. Our participants, especially the drug dispensers perceived that supplier incentives to the prescribers, is one of the factors responsible for higher antibiotic prescription rate. A study in China [32], in addition to the financial incentives to the prescribers, also identified fee-for-service treatment as a main driver of antibiotic overuse. This was not mentioned by our interviewees.

### Antibiotic use in dairy animals

Studies have documented the presence of antibiotics in milk from dairy animals [33,34]. The widespread use of antibiotics in dairy animals might also lead to the emergence of antibiotic resistant bacterial strains in their milk [35], something our participants also perceived as possible.

### Ineffective law enforcement

In India, state governments have adopted central government guidelines on policies and programmes for healthcare development, and the state of Orissa adopted a policy on rational drug use in 1997 [10]. Previous studies in India and other countries found that ineffective law enforcement increased the availability of fake or substandard medicines [36] and also availability of antibiotics without authorized prescription [26,37,38]. Our interviewees perceived such an issue to be existent in Orissa. A study from nine African countries suggests that, since most people purchase their drugs from drug dispensers due to lack of healthcare facilities, educating drug dispensers about rational drug use would probably be beneficial for public health in low-income countries [39]. It is likely that enforcement of the law forbidding sale of antibiotics without a prescription from a qualified allopathic prescriber would reduce the total amount of antibiotics used. Awaiting a situation where there are enough qualified prescribers to enforce the legislation fully, education for all types of providers (as they are anyway prescribing antibiotics) would be a way to decrease the improper use of antibiotics. Our interviewees also suggest that a law dissuading the use of antibiotics and promoting the use of probiotics as a growth promoter in poultry is also needed.

After the United Nations Conference on the Human Environment in Stockholm in 1972, India passed the Environmental (Protection) Act in 1986. It provides for "the protection and improvement of environment and the prevention of hazards to human beings, other living creatures, plants and property". There is need for effective enforcement of this law to protect our environment from pharmaceutical waste.

### Methodological consideration

A qualitative approach is the preferred method to explore an emerging concept **- **in this case the relationship between antibiotic use, resistance development and the environment. In order to improve the trustworthiness of our study, we used data triangulation during the analysis. Data was collected from different healthcare professionals including allopathic doctors, veterinarians and drug dispensers. The informants were from different geographical areas and socioeconomic environments, in this case proximity to sea/inland areas and urban/rural environments. During the coding procedure, both Oriya and English versions of the transcripts, and in some complex cases, tape recorded data, were used simultaneously in order to avoid misinterpretation of the full meaning. The authors of this study were from different educational backgrounds and countries, and each one brought a unique perspective to the study, enhancing its conformability. In the analyses, this factor served to broaden the interpretation and the final result is a negotiated outcome.

## Conclusions

Generally, the interviewees perceived that although behavioural and social environmental factors are major contributors to resistance development, changes in the physical and natural environment also influence development of antibiotic resistance through the occurrence of infectious diseases and consequent use of antibiotics. The authors feel that quantitative studies are needed to verify the perceptions of our interviewees regarding the interrelationships between environmental factors and antibiotic use and development of resistance. The authors have therefore initiated studies towards this end. The respondents also perceived that there is a lack of information about, and poor awareness of, what constitutes prudent use of antibiotics. They suggested a need for information, education, dissemination and proper implementation and enforcement of legislation at all levels of the drug delivery and disposal system in order to improve antibiotic use and prevent pharmaceutical contamination of the environment.

## Competing interests

The authors declare that they have no competing interests.

## Authors' contributions

KCS, AJT and CSL designed the study. KCS interviewed, transcribed and translated the interview transcripts from Oriya to English and made preliminary analysis. All authors contributed to the final analysis of the interviews. All authors have read and approved the final manuscript.

### Appendix 1

#### Interview guide including introductory questions and probing areas

1. What are the common indications of antibiotic prescribing/dispensing and factors influencing these? Probe: Common infectious diseases, antibiotics prescription and prior treatments

2. What is your view on antibiotic resistance? Probe: Possible causes, drug quality, human and nonhuman use of antibiotics and their impact

3. What is your view on environmental issues in relation to antibiotics? Probe: Antibiotic use and resistance development in relation to the environment, disposal of pharmaceutical waste and its impact

## Pre-publication history

The pre-publication history for this paper can be accessed here:

http://www.biomedcentral.com/1471-2458/10/629/prepub
